# Reply: Glasgow Prognostic Score as a predictive factor differentiating surgical site infection and remote infection following colorectal cancer surgery?

**DOI:** 10.1038/sj.bjc.6605354

**Published:** 2009-10-06

**Authors:** L Moyes, D McMillan

**Affiliations:** 1 University Department of Surgery, Faculty of Medicine-University of Glasgow, Royal Infirmary, Glasgow G31 2ER, UK

**Sir,**

We find the letter of Miki *et al* most interesting. As they state postoperative infectious complications can be separated into surgical site infections and remote infections. A surgical site infection such as a wound infection, anastomotic leak or intra-abdominal collection is an infection that occurs after surgery and is associated specifically with the surgical procedure. Surgical site infections can be further classified into incisional and organ/space infections. A remote infection such as pneumonia is often exogenous and occurs at sites not directly associated with the surgical procedure. In our paper, we have shown that the modified Glasgow Prognostic Score (mGPS) can predict postoperative infectious complications. Miki *et al* pose the question can the mGPS predict site-specific patterns in infectious complications? In particular, is an elevated mGPS associated with a greater proportion of patients with a remote infection?

In our cohort of 455 patients undergoing potentially curative colorectal resections, 70 patients developed an infectious complication. There were 31 surgical site infections (14 anastomotic leak, 2 intra-abdominal collections and 15 wound infections) and 39 remote infections (6 septicaemia, 33 pneumonia). In those patients with an infectious complication an elevated mGPS was not associated with a greater proportion of patients with a remote infection (*P*=0.899). Clearly, the numbers of surgical site infections and remote infections are relatively small and a larger cohort would need to be studied to exclude the possibility of the relationship posed by Miki *et al*.

Nevertheless, these results suggest that systemic inflammation influences both surgical site infections and remote infections. A plausible explanation for this, as outlined in our paper, is that the systemic inflammatory response is associated with compromised immune function.

